# Nanoparticle-Based Approaches for Enhancing In Vitro Fertilization in Animal Reproduction

**DOI:** 10.3390/ijms27062747

**Published:** 2026-03-18

**Authors:** Elżbieta Gałęska, Alicja Kowalczyk, Marko Samardžija, Gordana Gregurić Gračner, Marcjanna Wrzecińska, Jose Pedro Araujo, José Ángel Hernández Malagón, Mercedes Camiña, Ewa Czerniawska-Piątkowska, Zbigniew Dobrzański

**Affiliations:** 1 Department of Environment Hygiene and Animal Welfare, Wrocław University of Environmental and Life Sciences, 51-630 Wroclaw, Poland; elzbieta.galeska@upwr.edu.pl (E.G.);; 2 Faculty of Veterinary Medicine, University of Zagreb, Heinzelova 55, 10000 Zagreb, Croatia; smarko@vef.unizg.hr (M.S.); ggracner@vef.unizg.hr (G.G.G.); 3 Escola Superior de Agraria de Ponte de Lima, Instituto Politecnico de Viana do Castel, Refóios do Lima, 4990-706 Ponte de Lima, Portugal; 4 COPAR (GI-2120) Research Group, Department of Animal Pathology, Faculty of Veterinary, Campus Terra, University of Santiago de Compostela, 27002 Lugo, Spain; joseangel.malagon@usc.es; 5 Department of Physiology, Faculty of Veterinary Medicine, University of Santiago de Compostela, 27002 Lugo, Spain; 6 Department of Ruminant Science, West Pomeranian University of Technology in Szczecin, 70-310 Szczecin, Poland

**Keywords:** nanoparticles, reproduction, sperm, oocyte, nanosorting, nanopurification

## Abstract

Nanotechnology, based on nanoparticles, has become an emerging interdisciplinary tool in reproductive biotechnology, offering innovative opportunities to improve fertilization efficiency and reproductive performance in farm animals. The purpose of this review is to provide an updated synthesis of current research on nanoparticle-based approaches that enhance in vitro fertilization outcomes and other assisted reproductive technologies. The focus is on the biological mechanisms, potential benefits, and limitations of nanoparticle use in animal reproduction. Nanoparticles—including gold, silver, zinc oxide, selenium, and magnetic iron oxide—exhibit distinctive physicochemical properties that enable targeted interactions with gametes and reproductive cells. When used in semen extenders or culture media, nanoparticles improve sperm motility, acrosome and membrane integrity, and reduce oxidative stress and apoptosis. These effects contribute to enhanced fertilization rates and higher embryo developmental competence. In addition, nanoparticles can function as carriers for hormones, antioxidants, and growth factors, stabilizing reagents essential for oocyte maturation, sperm capacitation, and early embryo culture. The review also discusses nanopurification (selectively isolating and removing particles) and nanosorting (separating or organizing nanoscale objects) techniques that allow for non-invasive selection of viable gametes, and fluorescence- and magnet-assisted sorting systems that increase precision in sperm sexing. The mechanical aspects of nanoparticle–cell interactions are analyzed, emphasizing the influence of particle size, dose, and surface modification on both biological efficacy and cytotoxicity. Safety, toxicological concerns, and regulatory frameworks—including International Organization for Standardization (ISO) standards and European Commission recommendations—are critically reviewed to highlight the need for harmonized biocompatibility criteria. Although nanoparticle use in animal reproduction remains largely experimental, accumulated evidence demonstrates its potential to improve reproductive efficiency and reduce economic losses. Integrating nanoparticle-based systems with existing reproduction platforms may represent a transformative step toward sustainable and precision-driven livestock breeding.

## 1. Introduction

According to estimates by the Food and Agriculture Organization of the United Nations, to meet the needs of the growing world population, food production should increase by approximately 70% between 2030 and 2050. However, environmental constraints limit further expansion of livestock production, highlighting the need to improve reproductive efficiency rather than herd size [1,2]. 

In this context, nanoparticle-based approaches have emerged as promising tools for improving the efficiency of assisted reproductive technologies (ARTs), particularly in in vitro fertilization (IVF) [3].

Nanoparticles (NPs), unlike macroscopic particles, exhibit distinct physicochemical properties such as increased surface reactivity, altered polarity, specific electrostatic charge, and the ability to penetrate biological barriers. These properties are applicable in fields such as cellular and molecular biology, animal genetics, reproductive biotechnology, and veterinary physiology [4,5]. Although currently available data on the application of nanoparticles in animal production is scattered and heterogeneous, this topic has attracted significant interest. Research on the potential use of nanoparticles in veterinary medicine and animal husbandry is particularly intensive, especially in the area of assisted reproduction and reproductive control [6,7,8,9].

Additionally, NPs are used as carriers in targeted therapeutic systems, preclinical nanomodels, controlled drug release systems (accelerated and decelerated), and in selective therapeutic strategies aimed at limiting or enhancing the action of specific substances. These particles also exhibit enhanced permeability and retention, allowing them to preferentially accumulate in specific tissues [9].

**Figure 1:**
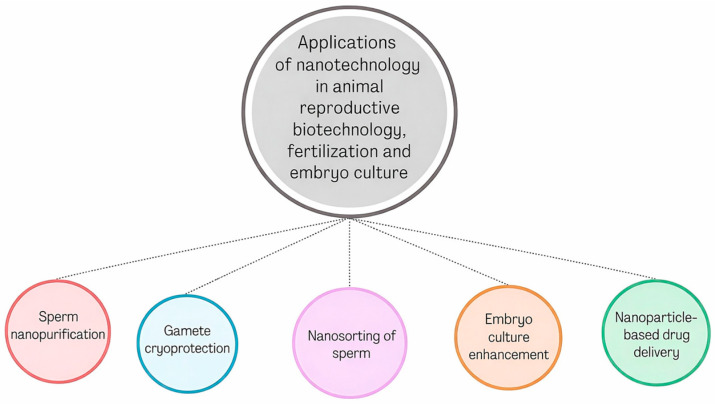
Applications of nanoparticles in animal in vitro fertilization [11]. Legend: Schematic overview of nanoparticle-based applications directly relevant to animal in vitro fertilization. Nanoparticles are used for sperm nanopurification, nanosorting, protection during cryopreservation, targeted delivery of antioxidants or hormones, and improvement of embryo developmental competence. These approaches aim to enhance fertilization efficiency and embryo quality while minimizing mechanical and oxidative damage to gametes.

In animal husbandry, the use of iron oxide (Fe_3_O_4_), zinc oxide (ZnO), and selenium (Se) is increasingly being indicated, partly due to their antioxidant properties. These substances can be used to synchronize estrus, protect frozen reproductive cells, and treat pregnancy complications [3,10]. Among modern, targeted therapies using nanoparticles, several main solutions stand out (Figure 1).

Significant limitations of NPs for routine use include poor chemical stability, low loading capacity, and the lack of biodegradability of certain materials, which is currently one of the most serious challenges in their biomedical applications. However, the more natural the compounds comprising nanomaterials, the more difficult they are to precisely modify and tailor for specific applications. Nevertheless, their high biocompatibility remains a key advantage that in many cases outweighs these limitations [12]. Further development in this field is closely linked to progress in related areas such as chemistry, bioengineering, and physics, technological development, and improvement of research funding systems [12,13].

Importantly, this review focuses exclusively on experimental and applied studies in which nanoparticles are directly used to enhance fertilization, sperm selection, gamete protection, or embryo development in animals. Broader discussions of ART, animal physiology, or agricultural management are intentionally omitted to maintain a strict focus on nanoparticle-based IVF strategies.

## 2. Nanoparticles in Assisted Reproductive Technologies

In reproductive biotechnology, only selected classes of nanoparticles have demonstrated practical applicability in IVF-oriented systems. Among them, magnetic iron oxide nanoparticles (Fe_3_O_4_) are the most extensively studied due to their magnetic responsiveness, biocompatibility, and ease of surface functionalization. These properties enable their use in sperm selection and purification procedures without extensive mechanical stress [14,15,16].

Gold nanoparticles (AuNPs) have been investigated primarily as functionalizable carriers and molecular probes because of their high surface reactivity and chemical stability. Their capacity for conjugation with annexin V, lectins, and antibodies enables selective targeting of sperm surface markers associated with apoptosis, acrosomal damage, and ubiquitination. However, their biological effects are strongly dependent on particle size and concentration [17,18].

Antioxidant nanoparticles, including selenium nanoparticles (SeNPs) and cerium oxide nanoparticles (CeO_2_s), have been evaluated for their ability to reduce oxidative stress during semen processing and cryopreservation. In contrast, certain metallic nanoparticles such as silver nanoparticles (AgNPs) and high-dose zinc oxide nanoparticles (ZnOs) have demonstrated dose-dependent cytotoxic effects in gamete and embryo-related models, limiting their applicability in IVF settings [3,7,8,10,17,19].

The suitability of nanoparticles for reproductive applications is therefore determined by several key physicochemical parameters: particle size, surface charge, functionalization strategy, colloidal stability in biological media, and concentration-dependent toxicity. These properties directly influence membrane interaction, reactive oxygen species (ROS) generation, and overall gamete viability [20,21,22].

Among the nanoparticle-based approaches evaluated in animal IVF, nanopurification has emerged as the most experimentally validated technique and is discussed in detail in Section 3. A structured synthesis of experimentally validated nanoparticle systems evaluated in animal IVF models is provided in Table 1. The table summarizes nanoparticle type, surface modification strategy, applied dose, model species, and the observed biological outcomes, including sperm viability, cleavage rate, blastocyst formation, and pregnancy success. As shown in Table 1, magnetic Fe_3_O_4_ nanoparticles, functionalized AuNPs, and selected antioxidant nanoparticles such as SeNPs and CeO_2_s have demonstrated reproducible applicability in IVF-oriented systems.

The biological behavior of nanoparticles in IVF systems is determined primarily by physicochemical parameters that directly affect their interaction with gametes. In particular, particle size influences membrane penetration and cellular uptake; surface charge modulates electrostatic interactions with sperm membranes; and functionalization strategies determine binding specificity toward selected molecular markers [18,20,23]. In addition, colloidal stability in biological media and strict dose optimization are essential to avoid unintended ROS generation or cytotoxic effects [21,24].

**Table 1:** Experimental studies evaluating nanoparticles for improving animal in vitro fertilization and semen processing.

Nanoparticle Type	Functionalization/Application	Animal Model	Dose/Concentration	Experimental Endpoint (IVF-Related)	Key Outcome	Ref.
AuNPs	Added to semen extender	Bovine	10 µg/mL	Sperm motility, apoptosis, IVF outcome	↓ apoptosis, ↑ motility, ↑ pregnancy rate (~30%)	[20]
Fe_3_O_4_ NPs	Lectin- or annexin V–conjugated (nanopurification)	Bovine	Not specified	Removal of apoptotic and acrosome-damaged sperm	↑ viable sperm fraction, ↑ IVF efficiency	[23]
ZnO NPs	Supplemented during semen processing	Bovine	50 µg/mL	Blastocyst formation rate	↑ blastocyst yield without embryotoxicity	[6]
Se NPs	Added to semen extender	Bovine	1 µg/mL	Post-thaw sperm viability and motility	↑ post-thaw viability, ↓ oxidative stress	[3]
CeO_2_ NPs	Short-term sperm exposure	Ram	220 µg/mL	Sperm viability and morphology	No toxicity, ↑ sperm viability	[10]
Cooper nanoparticles (Cu NPs)	Semen extender supplementation	Bovine	0.7–1.0 µg/mL	DNA integrity after cryopreservation	↓ DNA fragmentation	[25]
Ag NPs	Added to oocyte maturation medium	Mouse	125 µg/mL	Oocyte maturation, embryo development	↓ maturation rate (dose-dependent toxicity)	[7]
AuNPs	Added to oocyte activation medium	Mouse	250 nM	Morula and blastocyst development	↑ morula and blastocyst formation	[26]
Fe_3_O_4_ NPs	Magnetic sperm sorting	Horse	Not specified	IVF pregnancy rate	~80% pregnancy rate, female-biased offspring	[27]
ZnO NPs	Semen supplementation	Bovine	20–50 µg/mL	ROS levels, embryo development	↓ ROS, ↑ embryo quality	[8]

Therefore, the applicability of a given nanoparticle class in reproductive biotechnology is not defined solely by its chemical composition, but by the controlled integration of structural design, surface modification, and concentration-dependent safety. These determinants explain why only selected nanoparticle systems have demonstrated reproducible benefits in IVF-oriented models [9,18].

**Figure 2:**
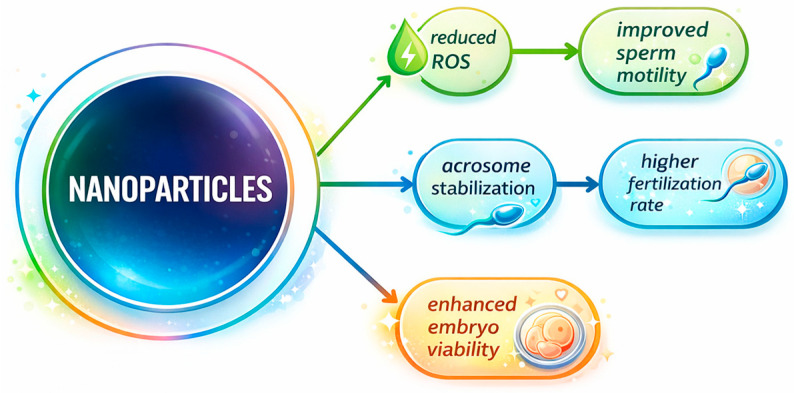
Mechanisms of nanoparticle action in in vitro fertilization [28]. Legend: Molecular and cellular mechanisms through which nanoparticles influence animal in vitro fertilization. Nanoparticles may (i) reduce oxidative stress by scavenging reactive oxygen species, (ii) protect sperm membranes and acrosomes, (iii) selectively bind and remove defective spermatozoa during nanopurification, and (iv) enhance embryo development by stabilizing culture conditions.

The biological effects of nanoparticles on gametes and embryos are mediated through multiple mechanisms, including oxidative stress reduction and selective sperm removal (Figure 2).

## 3. Nanopurification and Nanosorting in Animal IVF

Nanopurification is a nanoparticle-based sperm selection strategy designed to improve the quality of gametes used for in vitro fertilization. The technique relies primarily on functionalized magnetic iron oxide nanoparticles (Fe_3_O_4_s), which selectively bind spermatozoa exhibiting molecular markers of apoptosis, membrane destabilization, acrosomal damage, or ubiquitination. Nanoparticles are typically conjugated with annexin V, lectins such as peanut agglutinin, or antibodies targeting ubiquitinated proteins. After incubation, nanoparticle-bound sperm are removed using a magnetic field, yielding a purified fraction enriched in fertilization-competent cells [18,20,23].

Experimental studies in cattle and other livestock species have demonstrated that nanopurified semen exhibits higher post-thaw viability, improved mitochondrial activity, and increased progressive motility compared with unpurified controls [7,18]. Importantly, IVF performed using nanopurified sperm has been associated with increased cleavage rates, improved blastocyst formation, and higher pregnancy rates following embryo transfer [20]. These experimentally confirmed outcomes, including improvements in post-thaw motility, cleavage rate, blastocyst yield, and pregnancy success across different animal models, are summarized in Table 1.

Compared with conventional sperm selection techniques such as density gradient centrifugation or swim-up, nanopurification reduces mechanical stress and limits ROS generation during processing. This distinction is particularly relevant for IVF, where oxidative damage to sperm deoxyribonucleic acid (DNA) and membranes negatively affects embryo quality [29].

Nanosorting represents a related but conceptually distinct application of nanoparticles. In contrast to nanopurification—which primarily removes defective sperm—nanosorting aims to isolate specific sperm subpopulations based on molecular or surface characteristics. Magnetic and gold nanoparticles have been investigated for sperm sex selection and targeted subpopulation enrichment. Although promising results have been reported, nanoparticle-assisted sperm sexing remains at the experimental stage and has not yet demonstrated sufficient robustness or scalability to replace conventional flow cytometry-based methods [30,31].

**Figure 3:**
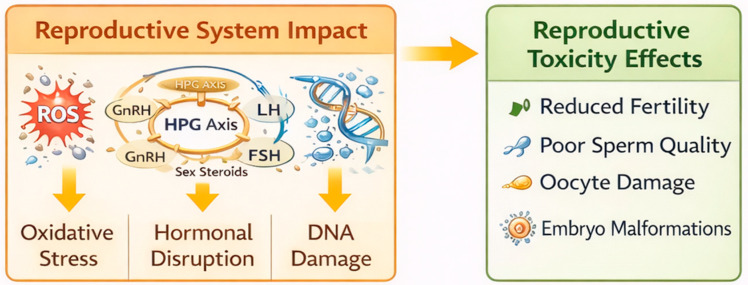
Schematic representation of the mechanisms through which nanoparticles may affect the reproductive system. Legend: Nanoparticle-induced oxidative stress (ROS), disruption of the hypothalamic–pituitary–gonadal (HPG) axis, and DNA damage are illustrated as key biological pathways potentially leading to reduced fertility, impaired sperm quality, oocyte damage, and embryo malformations.

Oxidative stress, membrane destabilization, and cryopreservation-induced damage remain major factors (Figure 3) limiting sperm viability and fertilization efficiency under in vitro conditions. Nanoparticles have therefore been explored as auxiliary tools to protect gametes during semen processing and culture by reducing reactive oxygen species and stabilizing membrane structures. Importantly, these effects are highly dependent on nanoparticle type, surface modification, and dose, emphasizing the need for controlled experimental validation rather than generalized application [21,24].

Fluorescence-based assays are primarily used as validation tools to assess the effectiveness of nanoparticle-based sperm purification rather than as independent reproductive technologies. In experimental studies, fluorescent probes are employed to confirm selective binding of nanoparticles to damaged spermatozoa and to quantify improvements in sperm quality following nanopurification [31,32,33].

For example, lectins conjugated with fluorescein isothiocyanate (FITC), such as peanut agglutinin (PNA), selectively bind exposed glycosidic residues on damaged acrosomes. When used in combination with magnetic nanoparticles, fluorescence microscopy or flow cytometry confirms the efficient removal of acrosome-compromised sperm from semen samples [31,34]. These validation approaches provide mechanistic confirmation that nanoparticle-mediated selection reduces the proportion of apoptotic and structurally compromised spermatozoa in the fertilization medium [31,32,33].

## 4. Molecular and Cellular Mechanisms Affected by Nanoparticles

### 4.1. Gamete Quality Enhancement

Certain NPs have attracted more attention than others, particularly due to their stability and compatibility. Fe_3_O_4_ is one such particle that appears to be suitable for use in reproduction. These particles, when added to buffers, have a positive effect on sperm, providing protection against microorganisms [35]. In the context of reproductive biotechnology, however, the functional relevance of nanoparticles is determined primarily by their interactions with gametes and embryo culture environments rather than by their industrial or catalytic applications.

### 4.2. Targeted Delivery System

The possibility of using special forms of AuNPs as biomarkers has been observed for over a decade. Complexes of such biomarkers with magnetic properties could potentially be used in semen nanopurification due to specific proteins on the sperm surface, which can be captured by NPs. In this case, they would act as probes specifically binding to structures on the surface of cell membranes. These proteins are highly specific to damage or other abnormalities in the cell, hence the possibility of targeted binding to NPs [36]. Proteins specific to damage or other abnormalities in sperm include phosphatidylserine (a marker of early apoptosis), annexin V (as an indirect marker related to phosphatidylserine), heat shock and lipid peroxidation-related proteins (markers of oxidative stress), Fas and Bax proteins (markers of apoptosis), ubiquinated proteins, and acrosomal proteins (markers of premature acrosomal reaction) [37,38]. AuNPs are linked via hybridization, which could also allow for simultaneous sexing of sperm by binding to unique proteins characteristic of the male Y chromosome. Such sorting could contribute to the development of ART and accelerate semen quality assessment [36]. Therefore, analytical techniques such as fluorescence microscopy or cytometric validation are discussed here solely in the context of confirming nanoparticle-mediated selection efficiency, rather than as independent reproductive technologies. It should be emphasized that magnetic nanoparticle-assisted sperm sexing remains at an experimental stage. Although promising, this method requires further validation regarding safety, reproducibility, and potential impacts on sperm integrity before being considered a viable alternative to flow cytometry-based sexing [31,34].

**Figure 4:**
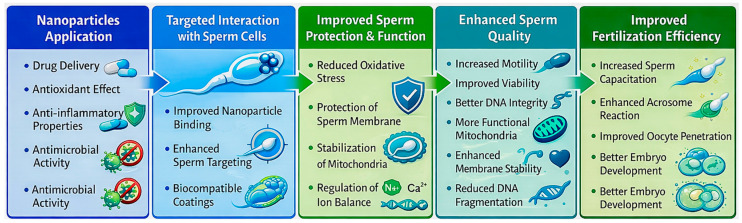
Nanoparticle–sperm interactions. Legend: Interaction between nanoparticles and spermatozoa during nanopurification and nanosorting. Functionalized nanoparticles selectively bind sperm exhibiting apoptotic markers, acrosome damage, or membrane destabilization. Magnetic separation enables removal of defective cells, enriching the fertilization-competent sperm population for IVF.

The broader spectrum of nanoparticle–sperm interactions relevant to IVF is schematically presented in Figure 4. Not only are economic and welfare concerns paramount, but also ecological, as extracts of natural origin can be used to synthesize ultra-small AuNPs [39]. These particles also exhibit antimicrobial and protective properties [40,41,42]. Selenium and zinc NPs are also gaining interest. The *in ovo* introduction of these particles has been shown to reduce oxidative stress and mitigate the effects of heat stress. This was achieved by simultaneously increasing the activity of glutathione peroxidase (GSH-Px) and superoxide dismutase (SOD), and reducing the levels of cortisol, corticosterone, triiodothyronine, and thyroxine [43]. Research also shows interest in introducing nanostructures into animal nutrition [44] and animal products themselves [45], indicating a much wider application of NPs in animal breeding.

The performance of nanoparticle-assisted sperm processing is determined primarily by nanoparticle physicochemical parameters, including surface functionalization, colloidal stability in biological media, and dose. Since the mechanistic principles of selective binding and magnetic separation are described in detail in Section 3, this subsection focuses on factors that modulate binding specificity and safety during IVF-related handling, rather than on analytical control methods.

The principle of nanoparticle-mediated sperm selection is illustrated in Figure 4.

### 4.3. Nanoparticle-Associated Molecular Strategies in IVF

The composition of the fluid used for *in vitro* maturation procedures has a huge impact on the quality of cells obtained. Proteins, cytokines, and growth factors all impact the chances of obtaining an embryo [46]. Standard IVF protocols include reagents crucial for gamete and embryo development. These include capacitation factors (heparin, body surface area (BSA), caffeine), oocyte maturation factors (follicle-stimulating hormone (FSH), epidermal growth factor (EGF), luteinizing hormone (LH)), and culture media supplements (amino acids, pirucan, and serum substitutes). Nanoparticles can increase the stability of these reagents or improve their targeted delivery. Gold or zinc NPs can be used as carriers of antioxidants or hormones, supporting sperm capacitation and oocyte maturation [5,7,47,48]. Complexes prove effective for obtaining zygotes. By surrounding the desired molecules in the complex, NPs can stabilize and improve the reaction rate [49].

NP complexes can protect oocytes during routine reactions with common reagents, improving the reagents and techniques used for IVF. Further research should focus on nanoformulated culture systems to improve embryo viability and developmental competence [50].

### 4.4. Nanoparticle-Cell Interactions

Supplementation of AuNPs at a concentration of 10 µg/mL to diluted semen significantly reduced the proportion of apoptotic spermatozoa (28.03 ± 0.35% vs. 44.47 ± 0.84% in the control group; mean ± SD). Additionally, improvements were observed in sperm viability (39.78 ± 0.94% vs. 32.67 ± 1.33%), acrosome integrity (52.40 ± 1.21% vs. 40.00 ± 1.00%), plasma membrane integrity (83.22 ± 1.02% vs. 75.00 ± 0.92%), and progressive motility (82.78 ± 0.87% vs. 72.78 ± 0.87%) (all values expressed as mean ± SD). Furthermore, in cows, a 30% increase in pregnancy rate was reported following AuNP supplementation [51]. The use of AuNPs has been reported to increase the chances of more embryos reaching the morula or blastocyst stage during culture in vitro [26]. The authors showed that mouse oocytes collected at the MII stage and subjected to artificial activation in an activation medium containing 10 mM strontium chloride supplemented with 250 nM AuNPs showed a significant improvement in the percentage of embryos reaching the morula (75%) and blastocyst (58%) stages compared to control embryos—61% for the morula and 42% for the blastocyst, respectively [26]. The possibility of effective use of magnetic NPs in sorting sperm in terms of sex and regularity is also emphasized, translating directly into the efficiency of cattle breeding [52]. However, like any other external particles introduced into cells, NPs can lead to adverse effects, such as organelle or DNA damage. Conflicting data regarding the cytotoxicity of AuNPs appear in the literature [53]. Today, however, it is known that the danger of a particle depends on its size, charge and shape [28,54]. Several studies indicate that biological effects of nanoparticles are strongly dose-dependent, and that optimized concentration ranges are essential to preserve their beneficial properties while minimizing cytotoxicity. The biological response to nanoparticles depends on prior exposure history, species-specific sensitivity, and cumulative dose, which may influence immunological and oxidative stress pathways [28].

Nanotechnologies have developed with the addition of many additional processes, such as the swimming method (separation of progressively motile sperm), Sephadex filtration and glass wool filtration (aggregation of immobile sperm based on surface charges), and gradient separation using Percoll (evaluation of motility depending on density) [55,56,57]. These cell-based methods routinely utilize magnetic particles. The most frequently mentioned in the literature is Fe_3_O_4_. For these analyses, researchers often use the molecules annexin V and lectin. The former allows for the detection of apoptosis, while the latter acts as a detector of acrosome damage. Combining these elements may allow for effective cell selection in bovine semen [58]. In contrast to conventional sex-sorting approaches, nanoparticle-assisted strategies aim to improve fertilization outcomes primarily through enhancement of sperm functional integrity rather than direct chromosomal separation [32,33].

## 5. Safety, Toxicity and Regulatory Issues

Among the existing regulations, several important normative documents can be identified, applicable to the characterization, risk assessment and certification of nanomaterials [58]. In terms of general standards, the following deserve special attention:

- ISO 27687 [59]—defines basic concepts related to nanomaterials, including the distinction between NPs, nanofibers and nanoplatforms;
- ISO 21083 [59]—defines the methodology for testing the effectiveness of filter materials with respect to NPs in the size range of 20–500 nm [59].

In the context of biomedical applications, the following standard is important:

- PN-EN ISO 10993 [60]—encompassing a comprehensive set of methods for assessing the biocompatibility of nanomaterials. This includes, among others, cytotoxicity, hemolysis and thrombogenicity testing, as well as assessing the impact of sterilization and biodegradation processes on materials in contact with biological systems [60].

Safety assessment and certification procedures for nanomaterials are carried out on the basis of:

- ISO/TS 17200:2013(E) [61]—a technical specification containing guidance on the identification and characterization of nanomaterials in the environment and their potential health impacts;
- 2022/C 229/01 [61]—the European Commission recommendations, which specify the definition of a nanomaterial and the criteria for its classification in the context of European chemical and sanitary law [61].

One of the main reasons for the limited effectiveness of antioxidants in clinical trials for inflammatory diseases is their low capacity to eliminate ROS when used as single antioxidant compounds [9]. Research results indicate that the cytotoxicity of NPs depends significantly on their size—the smaller the particle diameter, the greater the risk of toxicity during interaction with cells. This phenomenon stems from physical factors: reducing the particle diameter leads to an increased surface-to-volume ratio, which intensifies its chemical reactivity. Furthermore, smaller NPs more easily penetrate difficult-to-access intracellular structures, such as mitochondria or the cell nucleus which may intensify their toxic effects. The mechanism of cytotoxicity is highly complex, as it depends on numerous factors. Its initial stage is direct or indirect cell penetration. This is followed by binding and accumulation within active structures (lysosomes, mitochondria, cell nuclei). During toxic reactions, nanoparticles generate reactive oxygen species and oxidative stress, damage the cell membrane and disrupt organelle function. The intensity of nanoparticle toxicity can be determined based on the extent of the activation of negative metabolic pathways [62].

Chronic exposure of cattle to heavy metals and other toxic trace elements can cause embryotoxicity, impaired spermatogenesis and abnormalities in oocyte development [63,64]; therefore, it is crucial to regularly monitor their levels in the surroundings of farms [65] in order to maintain productivity and conception potential while introducing new innovative solutions [66].

The question arises as to what the appropriate dose is for this type of process. NPs are more reactive, so they can pose a toxic threat more quickly [28]. NPs have been analyzed repeatedly to obtain information about their adverse effects. Silver NPs placed in semen extender at concentrations of 125 and 250 µg/mL were tested and routine cryopreservation was performed. Increased motility, decreased lipid peroxidation and reduced ROS levels were observed even at lower concentrations. Meanwhile, microbial load was within normal limits [67]. In the case of gold NPs at concentrations of 1.56 and 3.125 mg/L and copper NPs at concentrations of 12.5 and 25 mg/L, biocidal properties were observed [68]. In the low dose range, further research is particularly recommended, both in the context of animal health and the ecosystem [69]. It is difficult to discuss specific toxicity values and safe doses. Research into optimal doses for achieving specific goals is still intensive. However, it can be stated with certainty that the toxicity of a single nanoparticle will vary depending on the configuration, vector, and source used.

**Figure 5:**
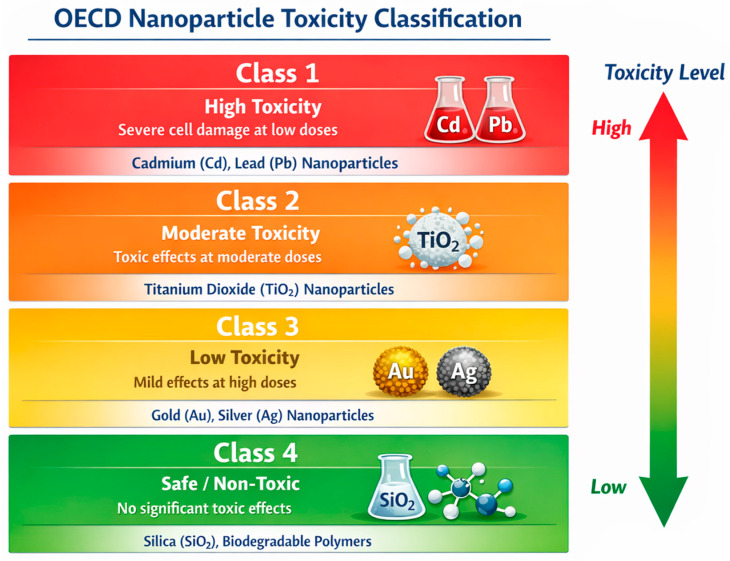
Material-based overview of nanoparticle toxicity categories. Legend: Nanoparticles are grouped according to reported toxicity profiles associated with their chemical composition: cadmium and lead (high toxicity), titanium dioxide (moderate toxicity), gold and silver (low toxicity), and silica or biodegradable polymers (minimal toxicity). This schematic illustrates material-dependent trends only.

The classification of nanoparticle toxicity (Figure 5) is important in nanotechnology and nanomedicine because the properties of nanoparticles (size, shape, surface, chemical composition) can influence their interactions with the body and the environment. The Organisation for Economic Co-operstion and Development (OECD) created a classification which determines general toxicity, as presented in Figure 5 [70].

Work on determining the optimal concentration of nanoparticles used in reproductive models began over a decade ago [71], particularly in response to their demonstrated ability to permeate cellular membranes and accumulate within cells [72]. Additionally, already in the early 2000s, the toxicity of nanoparticle precursors and metal-derived ionic forms was highlighted [73]. Subsequent studies therefore aimed to define not only the appropriate particle size for safe cellular interactions, but also concentration-dependent effects and interactions with biological structures present in reproductive systems. Gold nanoparticles measuring 15 nm in diameter exhibited no detectable toxic effects across the tested concentration range, whereas smaller particles induced necrosis and programmed cell death [53]. A decade later, the optimal size for these gold-containing particles was determined to be 25–50 nm. This size allows for appropriate reactivity, stimulation and interaction at the receptor–ligand level [74]. A few years later, the proper shape of these particles was also determined. At the same time, it was confirmed that even a size of 50 nm has antioxidant effects without increasing toxicity [54].

The effect of gold-containing NPs on immunoglobulin G (IgG) and γ-globulin antibodies was verified. No significant difference was found depending on the NP variant. However, it was observed that long-term exposure to NPs may result in certain adverse reactions, including increased IgG levels in downstream metabolic pathways [75]. It was shown that after repeated injection of gold NPs, the level of γ-globulin decreased and the transformation of lymphocytes increased compared to a single dose [54]. Researchers demonstrated that the use of AuNPs did not increase the level of proinflammatory cytokines and significantly reduced the level of tumor necrosis factor-alpha (TNF-α). An increase in the size of macrophages and a decrease in their number were also observed. Simultaneously, an increase in the production of interleukin (IL)-1 and IL-6 was demonstrated [76]. Despite these achievements, the method of measuring NPs still requires refinement. The biggest challenge seems to be reducing overestimation and the use of incorrect mathematical formulas [77]. Despite this, researchers are working hard to optimize processes related to NPs and medicine [78,79,80].

Concerns about the possible toxicity of AuNPs arise primarily from human health, and environmental concerns [12]. Silver NPs have been studied as a potentially better solution for animal reproduction. However, their accumulation in the fetal cumulus cells has been shown to lead to cell impairment and a slower development rate. Impairment of the oocyte maturation process itself has also been observed. This is most likely due to oxidation and is questionable in the context of verifying specific NPs, but the use of AuNPs appears to be a better solution [14].

## 6. Practical Application of Nanoparticles in Fertilization In Vitro and Future Prospects

The first serious problem with sorting techniques is still the lack of availability of such products in the world [81] and the quality of the insemination procedure itself, which can waste an excellent dose of sexed semen [82,83]. Sexed sperm have been shown to have a shorter lifespan than conventional semen, which indirectly impacts fertilization efficiency. Hormonal synchronization, preovulatory follicle size (POF) and heat stress are also problematic. Despite the quality of the sperm straw, all of these factors can negatively impact fertility and fertilization rates [84,85,86,87].

At the same time, as part of ART support activities, NPs were placed in calorimetric sensors and the possibility of more accurate identification of the occurrence of heat was tested, which turned out to be potentially effective [88]. It was shown that significantly more live sperm could be obtained in the case of nanopurified semen (65.68 ± 4.74%) compared to the unpurified fraction (46.58 ± 1.4%). At the same time, fewer dead sperm were observed in the nanopurified semen (22.21 ± 4.327%) compared to the unpurified semen (32.32 ± 4.44%) [18]. Researchers have recently shown a fertility rate for sexed semen of between 71.5 and 78.5% [89]. Using ablations for sexing has shown a 78% conception rate in beef cattle [84]. Immunosorting, on the other hand, produced a significantly higher percentage of female calves (74.29%) than conventional semen (47.22%), with no difference in pregnancy rates between the two methods [90]. In horses, sorting with magnetic NPs resulted in a nearly 80% pregnancy rate, with 95% of conceptions confirmed as female by ultrasound. Using sex-specific X factors coated on magnetic beads, they achieved an 80% (4/5) pregnancy rate in mares [27]. Ultimately, the implementation of highly accurate biomarker-based semen analysis, sperm selection, sperm sexing and semen storage methods, such as those discussed in this article, will further enhance the utility of artificial insemination and improve artificial insemination semen products across the industry [91,92,93,94].

In sheep, interesting results have also been obtained from studies using nanoparticles. They demonstrated reduced ROS levels, restored mitochondrial function, and potentially increased oocyte resistance to cryoinjury [95]. Improvements in pregnancy rates and live births were observed during short-term synchronized estrus. This increase in birth rates is due, among other things, to improved gamete quality, which could translate into overall production optimization [96]. Equally widespread use has been observed in poultry. However, the research expanded to include dietary and environmental issues. It was determined that the use of copper nanoparticles in feed not only improved growth and reduced the likelihood of disease, but also reduced the excretion of this element into the environment [97]. As mentioned, nanoparticles also find applications in biosecurity. Their properties allow them to remove antibiotic residues [98] and degrade 80–93% of the keratin in chicken feathers, which is also important in the breeding of these animals. The cycle closes with the initiation of research on the secondary products of these activities, which can serve as substrates for subsequent reactions with nanoparticles [99].

The efficiency of sperm sorting in animals is currently at a significant level, with studies conducted in many different animal species. In rams, the efficiency rate exceeds 92% [100]. Companies have also entered the market offering commercial kits containing nanoparticles for cattle, pigs, horses, and other animals. They report pregnancy prediction accuracy ranging from 70% (in sows) to 90% (in heifers) [81]. Detailed analyses have demonstrated that three independently evaluated nanoparticle-assisted semen preparation protocols resulted in increased pregnancy rates of 17.5%, 20%, and 30% compared with conventional semen controls [51]. The corresponding conception rates achieved in these experimental groups were 90%, 85%, and 82.5%, respectively, compared with 65% in the control group using conventional semen [51]. Flow cytometry remains the most accurate method of sperm sex sorting, with reported accuracies of ≥90% under controlled laboratory conditions and 70–90% in commercial applications, depending on semen type and processing method [81].

In contrast, nanoparticle-based approaches have primarily focused on improving sperm functional parameters, including membrane integrity, acrosome integrity, and morphology, which are directly associated with fertilization success. Reported improvements in conception rates vary between 5% and 25%, depending on species and experimental conditions [81,101,102]. However, further large-scale validation studies are required before these approaches can be considered fully standardized [103].

## 7. Conclusions

Nanoparticle-based strategies represent a promising but still predominantly experimental approach for improving in vitro fertilization outcomes in animal reproduction. Available evidence indicates that nanopurification using functionalized nanoparticles can enhance sperm quality and fertilization efficiency by selectively removing damaged or apoptotic cells, while minimizing the mechanical and oxidative stress associated with conventional selection methods. Other applications, including nanoparticle-assisted nanosorting and targeted delivery of bioactive compounds, remain at the proof-of-concept stage and require further validation. The biological effects of nanoparticles are strongly dependent on particle type, surface modification, and dose, highlighting the necessity for standardized experimental designs and rigorous safety assessment. Future progress in this field will depend on systematic, well-controlled studies that directly link nanoparticle properties to fertilization and embryo development outcomes, rather than on extrapolation from broader nanotechnological or reproductive applications.

## Data Availability

The raw data supporting the conclusions of this article will be made available by the authors on request.

## References

[1] Wanapat M., Suriyapha C., Dagaew G., Matra M., Phupaboon S., Sommai S., Pongsub S., Muslykhah U. (2024). Sustainable livestock production systems are key to ensuring food security resilience in response to climate change. Agric. Nat. Resour..

[2] Costa C., Wollenberg E., Benitez M., Newman R., Gardner N., Bellone F. (2022). Roadmap for achieving net-zero emissions in global food systems by 2050. Sci. Rep..

[3] Khalil W.A., El-Harairy M.A., Zeidan A.E.B., Hassan M.A.E. (2019). Impact of selenium nano-particles in semen extender on bull sperm quality after cryopreservation. Theriogenology.

[4] Feugang J.M., Rhoads C.E., Mustapha P.A., Tardif S., Parrish J.J., Willard S.T., Ryan P.L. (2019). Treatment of boar sperm with nanoparticles for improved fertility. Theriogenology.

[5] Hashem N.M., Sallam S.M. (2020). Reproductive performance of goats treated with free gonadorelin or nanoconjugated gonadorelin at estrus. Domest. Anim. Endocrinol..

[6] Jahanbin R., Yazdanshenas P., Rahimi M., Hajarizadeh A., Tvrda E., Nazari S.A., Mohammadi-Sangcheshmeh A., Ghanem N. (2021). In Vivo and In Vitro Evaluation of Bull Semen Processed with Zinc (Zn) Nanoparticles. Biol. Trace Elem. Res..

[7] Piri M., Mahdavi A.H., Hajian M., Nasr-Esfahani M.H., Soltani L., Vash N.T. (2024). Effects of nano-berberine and berberine loaded on green synthesized selenium nanoparticles on cryopreservation and in vitro fertilization of goat sperm. Sci. Rep..

[8] Alfattah M.A., Hassan M.A.E., Shehabeldin A.M., Omar M.E.A., El-Nile A.E., Sindi R.A., Abd El-Rafaa L.A., Abdelnour S.A. (2025). Humic–zinc nanoparticles enhance cryopreserved bovine sperm quality: Evidence from kinematic, oxidative and in silico analyses. Ital. J. Anim. Sci..

[9] Wang K., Lu X., Lu Y., Wang J., Lu Q., Cao X., Yang Y., Yang Z. (2022). Nanomaterials in Animal Husbandry: Research and Prospects. Front. Genet..

[10] Falchi L., Bogliolo L., Galleri G., Ariu F., Zedda M.T., Pinna A., Malfatti L., Innocenzi P., Ledda S. (2016). Cerium dioxide nanoparticles did not alter the functional and morphologic characteristics of ram sperm during short-term exposure. Theriogenology.

[11] Shivakumar N. (2024). Recent Advances in Biological Nanodevices and Biosensors: Insights into Applications and Technological Innovations. Malays. NANO Int. J..

[12] Ali A., Ijaz M., Khan Y.R., Sajid H.A., Hussain K., Rabbani A.H., Shahid M., Naseer O., Ghaffar A., Naeem M.A. (2021). Role of nanotechnology in animal production and veterinary medicine. Trop. Anim. Health Prod..

[13] Formiga F.R., Şenel S. (2021). Nanotechnology and Veterinary Drug/Vaccine Delivery. Pharm. Nanotechnol..

[14] Silva J.R.V., Barroso P.A.A., Nascimento D.R., Figueira C.S., Azevedo V.A.N., Silva B.R., Santos R.P.D. (2021). Benefits and challenges of nanomaterials in assisted reproductive technologies. Mol. Reprod. Dev..

[15] Joudeh N., Linke D. (2022). Nanoparticle classification, physicochemical properties, characterization, and applications: A comprehensive review for biologists. J. Nanobiotechnol..

[16] Ul Haq Z., Hamadani H., Khan A.A., Ganai A.M., Beigh Y.A., Gull Sheikh G., Farooq J., Ahmad Ganai I., Ahman S.M. (2023). Nanotechnology: Changing the World of Animal Health and Veterinary Medicine. Interaction of Nanomaterials with Living Cells.

[17] More A., Mahajan S., Dakre S., Anjankar N., More D. (2025). Advancing Assisted Reproduction Technology: The Role of Nanotechnology in Enhancing Fertility Treatments and Diagnostics. J. Pharm. Bioallied Sci..

[18] Nilendu P., Kumaresan A., Talluri T.R., Raval K., Elango K., Pradeep Nag B.S., Duraisamy R., Manimaran A. (2024). Lectin Functionalised Iron Magnetic Nanoparticle-Based Sperm Selection: A Potential Technique to Improve Bull Sperm Quality In Vitro. Reprod. Domest. Anim..

[19] Wang Y., Fu X., Li H. (2025). Mechanisms of oxidative stress-induced sperm dysfunction. Front. Endocrinol..

[20] Odhiambo J.F., DeJarnette J.M., Geary T.W., Kennedy C.E., Suarez S.S., Sutovsky M., Sutovsky P. (2014). Increased Conception Rates in Beef Cattle Inseminated with Nanopurified Bull Semen. Biol. Reprod..

[21] Cheng Q., Wei T., Farbiak L., Johnson L.T., Dilliard S.A., Siegwart D.J. (2020). Selective organ targeting (SORT) nanoparticles for tissue-specific mRNA delivery and CRISPR–Cas gene editing. Nat. Nanotechnol..

[22] Ribas-Maynou J., Novo S., Torres M., Salas-Huetos A., Rovira S., Antich M., Yeste M. (2022). Sperm DNA integrity does play a crucial role for embryo development after ICSI, notably when good-quality oocytes from young donors are used. Biol. Res..

[23] Feugang J.M., Liao S.F., Crenshaw M.A., Clemente H., Willard S.T., Ryan P.L. (2015). Lectin-Functionalized Magnetic Iron Oxide Nanoparticles for Reproductive Improvement. JFIV Reprod. Med. Genet..

[24] Nagai T. (2024). How I overcame problems in in vitro fertilisation of livestock animals. Reprod. Fertil. Dev..

[25] Abdel-Halim B., Moselhy W., Helmy N. (2018). Developmental competence of bovine oocytes with increasing concentrations of nano-copper and nano-zinc particles during in vitro maturation. Asian Pac. J. Reprod..

[26] Muhammad T., Jamal M., Ashraf M., Zafar N., Shahzadi S., Maqbool T., Hadi F., Amjad R. (2021). Gold nanoparticles improve the embryonic developmental competency of artificially activated mouse oocytes. Vet. Res. Forum.

[27] Morris L.H., De Haan T., Landriscina L.G., Wilsher S., Gibb Z. (2018). The Effects of Nanoparticle Semen Purification on Semen Quality Parameters in Stallions. J. Equine Vet. Sci..

[28] Abbasi R., Shineh G., Mobaraki M., Doughty S., Tayebi L. (2023). Structural parameters of nanoparticles affecting their toxicity for biomedical applications: A review. J. Nanopart. Res..

[29] Charles D.K., Lange M.J., Ortiz N.M., Purcell S., Smith R.P. (2024). A narrative review of sperm selection technology for assisted reproduction techniques. Transl. Androl. Urol..

[30] Kansotia K., Naresh R., Sharma Y., Sekhar M., Sachan P., Baral K., Pandey S.K. (2024). Nanotechnology-driven Solutions: Transforming Agriculture for a Sustainable and Productive Future. J. Sci. Res. Rep..

[31] Bisla A., Honparkhe M., Srivastava N. (2022). A review on applications and toxicities of metallic nanoparticles in mammalian semen biology. Andrologia.

[32] Corion M., Keresztes J., De Ketelaere B., Saeys W. (2022). In ovo sexing of eggs from brown breeds with a gender-specific color using visible-near-infrared spectroscopy: Effect of incubation day and measurement configuration. Poult. Sci..

[33] Preuße G., Porstmann V., Bartels T., Schnabel C., Galli R., Koch E., Oelschlägel M., Uckermann O., Steiner G. (2023). Highly sensitive and quick in ovo sexing of domestic chicken eggs by two-wavelength fluorescence spectroscopy. Anal. Bioanal. Chem..

[34] Odhiambo J.F., Sutovsky M., DeJarnette J.M., Marshall C., Sutovsky P. (2011). Adaptation of ubiquitin-PNA based sperm quality assay for semen evaluation by a conventional flow cytometer and a dedicated platform for flow cytometric semen analysis. Theriogenology.

[35] Prasad M., Kumar R., Ghosh M., Syed S.M., Chakravarti S. (2024). Nanotechnology Theranostics in Livestock Diseases and Management. Livestock Diseases and Management.

[36] Sutovsky P., Kennedy C.E. (2013). Biomarker-Based Nanotechnology for the Improvement of Reproductive Performance in Beef and Dairy Cattle. Ind. Biotechnol..

[37] Tvrda E., Gosálvez J., Sánchez R., Du Plessis S.S. (2025). Dynamics of Selected Apoptotic and Heat Shock Proteins in Cryopreserved and Vitrified Semen from Normozoospermic Men. J. Microbiol. Biotechnol. Food Sci..

[38] Li J., Zhao W., Zhu J., Wang S., Ju H., Chen S., Basioura A., Ferreira-Dias G., Liu Z. (2023). Temperature Elevation during Semen Delivery Deteriorates Boar Sperm Quality by Promoting Apoptosis. Animals.

[39] Aljohani F.S., Hamed M.T., Bakr B.A., Shahin Y.H., Abu-Serie M.M., Awaad A.K., El-Kady H., Elwakil B.H. (2022). In vivo bio-distribution and acute toxicity evaluation of greenly synthesized ultra-small gold nanoparticles with different biological activities. Sci. Rep..

[40] Abdalhamed A.M., Ghazy A.A., Ibrahim E.S., Arafa A.A., Zeedan G.S.G. (2021). Therapeutic effect of biosynthetic gold nanoparticles on multidrug-resistant *Escherichia coli* and *Salmonella* species isolated from ruminants. Vet. World.

[41] El-Maddawy Z., El-sawy A., Ashoura N., Aboelenin S., Soliman M., Ellakany H., Elbestawy A., El-Shall N. (2022). Use of Zinc Oxide Nanoparticles as Anticoccidial Agents in Broiler Chickens along with Its Impact on Growth Performance, Antioxidant Status and Hematobiochemical Profile. Life.

[42] Mekonnen G. (2021). Review on Application of Nanotechnology in Animal Health and Production. J. Nanomed. Nanotechnol..

[43] Shokraneh M., Sadeghi A.A., Mousavi S.N., Esmaeilkhanian S., Chamani M. (2020). Effects of in ovo injection of nano-selenium and nano-zinc oxide and high eggshell temperature during late incubation on antioxidant activity, thyroid and glucocorticoid hormones and some blood metabolites in broiler hatchlings. Acta Sci. Anim. Sci..

[44] Patra A., Lalhriatpuii M. (2020). Progress and Prospect of Essential Mineral Nanoparticles in Poultry Nutrition and Feeding—A Review. Biol. Trace Elem. Res..

[45] Eskandani M., Janmohammadi H., Mirghelenj S.A., Ebrahimi M., Kalanaky S. (2021). Effects of Zinc Nanoparticles on Growth Performance, Carcass Characteristics, Immunity, and Meat Quality of Broiler Chickens. J. Appl. Anim. Sci..

[46] Aguila L., Treulen F., Therrien J., Felmer R., Valdivia M., Smith L.C. (2020). Oocyte Selection for In Vitro Embryo Production in Bovine Species: Noninvasive Approaches for New Challenges of Oocyte Competence. Animals.

[47] Nemcova L., Rosenbaum Bartkova A., Kinterova V., Toralova T. (2023). Importance of Supplementation during In Vitro Production of Livestock Animals. Theriogenology.

[48] Hosseinmardi M., Siadat F., Sharafi M., Roodbari N.H., Hezavehei M. (2022). Protective effect of cerium oxide nanoparticles on human sperm function during cryopreservation. Biopreserv. Biobank..

[49] Xi H., Huang L., Qiu L., Li S., Yan Y., Ding Y., Zhu Y., Wu F., Shi X., Zhao J. (2024). Enhancing oocyte in vitro maturation and quality by melatonin/bilirubin cationic nanoparticles: A promising strategy for assisted reproduction techniques. Int. J. Pharm. X.

[50] Kumar A., Andonissamy J., Selokar N.L., Thakur N.S., Kk K., Aderao G.N., Mehta H., Kc N., Sarkar S., Naskar S. (2025). On the Evolution and Applications of Nanoparticles in Livestock Reproductive Biotechnology: A Comprehensive Review. BioNanoScience.

[51] Khalil W.A., El-Rais M.S., Hegazy M.M., Hassan M.A.E., El-Raghi A.A., El-Moghazy M.M. (2024). The Effect of Metallic Nanoparticles Supplementation in Semen Extender on Post-thaw Quality and Fertilizing Ability of Egyptian Buffalo (*Bubalus bubalis*) Spermatozoa. Biol. Trace Elem. Res..

[52] Barchanski A., Taylor U., Klein S., Petersen S., Rath D., Barcikowski S. (2011). Golden Perspective: Application of Laser-Generated Gold Nanoparticle Conjugates in Reproductive Biology. Reprod. Domest. Anim..

[53] Pan Y., Neuss S., Leifert A., Fischler M., Wen F., Simon U., Schmid G., Brandau W., Jahnen-Dechent W. (2007). Size-Dependent Cytotoxicity of Gold Nanoparticles. Small.

[54] Mehanna E.T., Kamel B.S.A., Abo-Elmatty D.M., Elnabtity S.M., Mahmoud M.B., Abdelhafeez M.M., Abdoon A.S.S. (2022). Effect of gold nanoparticles shape and dose on immunological, hematological, inflammatory, and antioxidants parameters in male rabbit. Vet. World.

[55] Lee H.L., Kim S.H., Ji D.B., Kim Y.J. (2009). A comparative study of Sephadex, glass wool and Percoll separation techniques on sperm quality and IVF results for cryopreserved bovine semen. J. Vet. Sci..

[56] Li S., Nguyen L., Xiong H., Wang M., Hu T.C.C., She J.X., Serkiz S.M., Wicks G.G., Dynan W.S. (2010). Porous-wall hollow glass microspheres as novel potential nanocarriers for biomedical applications. Nanomed. Nanotechnol. Biol. Med..

[57] Sinjari B., Pizzicannella J., D’Aurora M., Zappacosta R., Gatta V., Fontana A., Trubiani O., Diomede F. (2019). Curcumin/Liposome Nanotechnology as Delivery Platform for Anti-inflammatory Activities via NFkB/ERK/pERK Pathway in Human Dental Pulp Treated With 2-HydroxyEthyl MethAcrylate (HEMA). Front. Physiol..

[58] Bodu M., Ataman M.B., Bucak M.N., Öztürk A.E., Koca R.H., Akarsu S.A., Ömür A.D., Acısu T.C., Turhan A., Turhan A.B. (2023). Nanotechnology in the Purification of Semen. Nanotechnology in Reproduction.

[59] Jakubiak S. (2019). ISO 21083—New international standard for determination of nanoparticles filtration efficiency. Princ. Methods Assess. Walk. Environ..

[60] Wessely-Szponder J., Osmęcka D., Domańska A. (2024). Biomateriały Nadzieją Przyszłości.

[61] Brüngel R., Rückert J., Müller P., Babick F., Friedrich C.M., Ghanem A., Hodoroaba V., Mech A., Weigel S., Wohlleben W. (2023). NanoDefiner Framework and e-Tool Revisited According to the European Commission’s Nanomaterial Definition 2022/C 229/01. Nanomaterials.

[62] Kumar D., Mutreja I., Chitcholtan K., Sykes P. (2017). Cytotoxicity and cellular uptake of different sized gold nanoparticles in ovarian cancer cells. Nanotechnology.

[63] Akar Y., Ahmad N., Khalıd M. (2018). The effect of cadmium on the bovine in vitro oocyte maturation and early embryo development. Int. J. Vet. Sci. Med..

[64] Dutta S., Gorain B., Choudhury H., Roychoudhury S., Sengupta P. (2022). Environmental and occupational exposure of metals and female reproductive health. Environ. Sci. Pollut. Res..

[65] Wrzecińska M., Kowalczyk A., Cwynar P., Czerniawska-Piątkowska E. (2021). Disorders of the Reproductive Health of Cattle as a Response to Exposure to Toxic Metals. Biology.

[66] Lafontaine S., Labrecque R., Blondin P., Cue R.I., Sirard M.A. (2023). Comparison of cattle derived from in vitro fertilization, multiple ovulation embryo transfer, and artificial insemination for milk production and fertility traits. J. Dairy Sci..

[67] Kanwar A., Virmani M., Lal S., Chaudhary K., Kumar S., Magotra A., Pandey A.K. (2023). Silver nanoparticle as an alternate to antibiotics in cattle semen during cryopreservation. Anim. Reprod..

[68] Kalińska A., Wawryło C., Tlatlik W., Gołębiewski M., Kot M., Lange A., Jaworski S. (2024). Preliminary In Vitro Evaluation of Silver, Copper and Gold Nanoparticles as New Antimicrobials for Pathogens That Induce Bovine Locomotion Disorders. Int. J. Mol. Sci..

[69] Iavicoli I., Leso V., Fontana L., Calabrese E. (2018). Nanoparticle Exposure and Hormetic Dose–Responses: An Update. Int. J. Mol. Sci..

[70] Doak S.H., Andreoli C., Burgum M.J., Chaudhry Q., Bleeker E.A.J., Bossa C., Domenech J., Drobne D., Fessard V., Jeliazkova N. (2023). Current status and future challenges of genotoxicity OECD Test Guidelines for nanomaterials: A workshop report. Mutagenesis.

[71] Verma H.N., Singh P., Chavan R.M. (2014). Gold nanoparticle: Synthesis and characterization. Vet. World.

[72] Miranova T., Hadjiargyrou M., Simon M., Jurukovski V., Rafailovich M.H. (2010). Gold nanoparticles cellular toxicity and recovery: Effect of size, concentration and exposure time. Nanotoxicology.

[73] Connor E.E., Mwamuka J., Gole A., Murphy C., Wyatt M. (2005). Gold Nanoparticles Are Taken Up by Human Cells but Do Not Cause Acute Cytotoxicity. Communication.

[74] Bloise N., Massironi A., Della Pina C., Alongi J., Siciliani S., Manfredi A., Biggiogera M., Rossi M., Ferruti P., Ranucci E. (2020). Extra-Small Gold Nanospheres Decorated with a Thiol Functionalized Biodegradable and Biocompatible Linear Polyamidoamine as Nanovectors of Anticancer Molecules. Front. Bioeng. Biotechnol..

[75] May S., Hirsch C., Rippl A., Bohmer N., Kaiser J.P., Diener L., Wichser A., Bürkle A., Wick P. (2018). Transient DNA damage following exposure to gold nanoparticles. Nanoscale.

[76] Yen H., Hsu S., Tsai C. (2009). Cytotoxicity and Immunological Response of Gold and Silver Nanoparticles of Different Sizes. Small.

[77] Lizonova D., Nagarkar A., Demokritou P., Kelesidis G.A. (2024). Effective density of inhaled environmental and engineered nanoparticles and its impact on the lung deposition and dosimetry. Part. Fibre Toxicol..

[78] Nair A., Shah J., Al-Dhubiab B., Jacob S., Patel S., Venugopala K., Morsy M., Gupta S., Attimarad M., Sreeharsha N. (2021). Clarithromycin Solid Lipid Nanoparticles for Topical Ocular Therapy: Optimization, Evaluation and In Vivo Studies. Pharmaceutics.

[79] Ouyang B., Poon W., Zhang Y.N., Lin Z.P., Kingston B.R., Tavares A.J., Zhang Y., Chen J., Valic M.S., Syed A.M. (2020). The dose threshold for nanoparticle tumour delivery. Nat. Mater..

[80] Serrano D.R., Luciano F.C., Anaya B.J., Ongoren B., Kara A., Molina G., Ramirez B.I., Sánchez-Guirales S., Simon J.A., Tomietto G. (2024). Artificial Intelligence (AI) Applications in Drug Discovery and Drug Delivery: Revolutionizing Personalized Medicine. Pharmaceutics.

[81] Quelhas J., Pinto-Pinho P., Lopes G., Rocha A., Pinto-Leite R., Fardilha M., Colaço B. (2023). Sustainable animal production: Exploring the benefits of sperm sexing technologies in addressing critical industry challenges. Front. Vet. Sci..

[82] Chebel R.C., Cunha T. (2020). Optimization of timing of insemination of dairy heifers inseminated with sex-sorted semen. J. Dairy Sci..

[83] Diskin M.G. (2018). Review: Semen handling, time of insemination and insemination technique in cattle. Animal.

[84] Perry G.A., Walker J.A., Rich J.J.J., Northrop E.J., Perkins S.D., Beck E.E., Sandbulte M.D., Mokry F.B. (2020). Influence of Sexcel^TM^ (gender ablation technology) gender-ablated semen in fixed-time artificial insemination of beef cows and heifers. Theriogenology.

[85] Diniz J.H.W., Peres R.F.G., Teixeira A.C.B., Riveros J.A.N., Noronha I.M., Martins C.F.G., Oliveira C.S., Pohler K.G., Pugliesi G., Oliveira L.Z. (2021). Administration of PGF2α at the moment of timed-AI using sex-sorted or conventional semen in suckled nelore cows with different intensity of estrus behavior. Theriogenology.

[86] Orsolini M.F., Meyers S.A., Dini P. (2021). An Update on Semen Physiology, Technologies, and Selection Techniques for the Advancement of In Vitro Equine Embryo Production: Section II. Animals.

[87] Pfeifer L.F.M., Castro N.A., Melo V.T.O., Neves P.M.A., Cestaro J.P., Schneider A. (2015). Timed artificial insemination in blocks: A new alternative to improve fertility in lactating beef cows. Anim. Reprod. Sci..

[88] Manikkaraja C., Mahboob S., Al-Ghanim K.A., Rajesh D., Selvaraj K., Sivakumar M., Al-Misned F., Ahmed Z., Archunan G. (2020). A novel method to detect bovine sex pheromones using l-tyrosine-capped silver nanoparticles: Special reference to nanosensor based estrus detection. J. Photochem. Photobiol. B Biol..

[89] Joezy-Shekalgorabi S., Maghsoudi A., Mansourian M.R. (2017). Reproductive performance of sexed versus conventional semen in Holstein heifers in various semiarid regions of Iran. Ital. J. Anim. Sci..

[90] Thongkham M., Thaworn W., Pattanawong W., Teepatimakorn S., Mekchay S., Sringarm K. (2021). Spermatological parameters of immunologically sexed bull semen assessed by imaging flow cytometry, and dairy farm trial. Reprod. Biol..

[91] Zuidema D., Kerns K., Sutovsky P. (2021). An Exploration of Current and Perspective Semen Analysis and Sperm Selection for Livestock Artificial Insemination. Animals.

[92] Akhtar M.F., Ma Q., Li Y., Chai W., Zhang Z., Li L., Wang C. (2022). Effect of Sperm Cryopreservation in Farm Animals Using Nanotechnology Animals. Animals.

[93] Neculai-Valeanu A.S., Ariton A.M. (2021). Game-Changing Approaches in Sperm Sex-Sorting: Microfluidics and Nanotechnology. Animals.

[94] European Commission (2023). Directorate General for Health and Food Safety. Kantar Public. Attitudes of Europeans Towards Animal Welfare: Report.

[95] Liu Q., Liu A., Liu Y., Li J., Bai J., Hai G., Wang J., Liu W., Wan P., Fu X. (2024). Hydroxyapatite nanoparticle improves ovine oocyte developmental capacity by alleviating oxidative stress in response to vitrification stimuli. Theriogenology.

[96] Shehabeldin A.M., Salama M.S., Omar M.E.A., AbdEl-Rafaa L.A., Ashour M.A., Hassan M.A.E., El-Shereif A.A., Abdelmegeid A., Shukry M., Elolimy A.A. (2025). Improving the breeding capabilities of short-term estrus synchronized Ossimi sheep using pregnant mare serum gonadotropin loaded chitosan-nanoparticles. Front. Vet. Sci..

[97] Sharif M., Aziz-ur Rahman M., Ahmed B., Abbas R.Z., Hassan F. (2020). Copper Nanoparticles as Growth Promoter, Antioxidant and Anti-Bacterial Agents in Poultry Nutrition: Prospects and Future Implications. Biol. Trace Elem. Res..

[98] El-Ghany W.A.A., Shaalan M., Salem H.M. (2021). Nanoparticles applications in poultry production: An updated review. World’s Poult. Sci. J..

[99] Rai S.K., Mukherjee A.K. (2015). Optimization for production of liquid nitrogen fertilizer from the degradation of chicken feather by iron-oxide (Fe_3_O_4_) magnetic nanoparticles coupled β-keratinase. Biocatal. Agric. Biotechnol..

[100] Gonzalez-Marin C., Lenz R.W., Gilligan T.B., Evans K.M., Gongora C.E., Moreno J.F., Vishwanath R. (2016). 191 SexedULTRA^TM^, A New Method of Processing Sex Sorted Bovine Sperm Improves Post-Thaw Sperm Quality and In Vitro Fertility. Reprod. Fertil. Dev..

[101] Nadri T., Towhidi A., Zeinoaladini S., Gholami D., Riazi G., Martínez-Pastor F. (2025). Impact of particle size of nanoliposomes on the biological response of frozen-thawed sperm in Holstein bulls. Cryobiology.

[102] Khalil W.A., Elkhamy S.A., Hegazy M.M., Hassan M.A.E., Abdelnour S.A., El-Harairy M.A. (2024). The cryoprotective effects of celastrol nanoemulsion on post-thawed attributes and fertilizing ability of cryopreserved buffalo semen. Vet. Res. Commun..

[103] El-Raghi A.A., Mohammed A.K., Elmorsy E.M., Hassan M.A.E., Essawi W.M., Mohamed S.S.A., Hassan A.M.E., Hassan S.A. (2026). Nanophytosomal delivery of resveratrol as an effective strategy for enhancing frozen–thawed bovine sperm quality. Cryobiology.

